# Consequences of the spilled gallstones during laparoscopic cholecystectomy: a systematic review

**DOI:** 10.1186/s13017-022-00456-6

**Published:** 2022-11-02

**Authors:** Paschalis Gavriilidis, Fausto Catena, Gianluigi de’Angelis, Nicola de’Angelis

**Affiliations:** 1 Department of Surgery, Royal Cornwall Hospitals NHS Trust, Treliske, Truro, TR1 3LJ Cornwall UK; 2 Department of General and Emergency Surgery, Bufalini Hospital, Cesena, Italy; 3 Gastroenterology and Endoscopy Unit, University Hospital of Parma, University of Parma, Parma, Italy; 4 Unit of Colorectal and Digestive Surgery, DIGEST Department, Beaujon University Hospital (AP-HP), University Paris Cité, Clichy, France

**Keywords:** Spilled, Lost, Gallstones, Laparoscopic cholecystectomy, Systematic review

## Abstract

**Introduction:**

Complications secondary to spilled gallstones can be classified in the category of disease of medical progress because prior to advent of laparoscopic cholecystectomy very few reports published on the topic. The aim of the present study was to investigate the predisposing factors and the complication rate of spilled gallstones during laparoscopic cholecystectomy over the past 21 years.

**Methods:**

Embase, Pubmed, Medline, Google scholar and Cochrane library were systematically searched for pertinent literature.

**Results:**

Seventy five out of 181 articles were selected including 85 patients; of those 38% were men and 62% women. The median age of the cohort was 64 years old and ranged between 33 and 87 years. Only 23(27%) of the authors reported the incident of spillage of the gallstones during the operation. Time of onset of symptoms varied widely from the second postoperative day to 15 years later. Ten of 85 patients were asymptomatic and diagnosed with spilled gallstones incidentally. The rest of the patients presented with complications of severe morbidity and almost, 87% of the patients needed to be treated with surgical intervention and 12% with US ± CT scan guidance drainage. Only one perioperative death reported.

**Conclusions:**

Symptomatic patients with lost gallstones present with severe morbidity complications and required mostly major surgical procedures. Therefore, standardisation of the management of spilled gallstones is needed urgently. Hospitals need to review their policy with audits and recommendations and clinical guidelines are needed urgently.

## Introduction

Since 1992, laparoscopic cholecystectomy accepted as a treatment of choice for symptomatic cholelithiasis by consensus statement from the National Institute of health conference [1]. It has been reported that the incidence rate of perforation of gallbladder during laparoscopic cholecystectomy (LC) ranges from 6 to 40% [2, 3]. The incidence rate of spillage of gallstones secondary to perforation reported 16% [4]. In addition, 16 to 50% of spilled stones remained un-retrieved [4, 5]. They may migrate in different regions and the reported complication rate varies from 0.08 to 0.3% [6]. However, most recent evidence reported that the incidence rate of complications of spilled gallstones may ranges from 0.04 to 19% [7]. The management of the spilled gallstones varies widely. Notably, studies which analyse complications of the LCs did not mention perforation of the gallbladder and spillage of stones as complication [8]. In addition, a study from the UK reported that only one fifth of the surgeons document spillage of the gallstones as a potential complication in the consent form. Moreover, only half of them in case of spillage and un-retrieved stones inform the patient. They are reluctant to do that because this may lead to unnecessary stress and repeated examinations for presumed complications of low risk [9]. However, most recent evidence demonstrated that gallbladder perforation and spillage of stones may lead to complications of severe morbidity. In particular, acute cases, older age, male sex, number of spilled stones more than 15 with diameter > 1.5 cm, pigment stones and perihepatic localisation are predicting factors for developing severe complications [3].

Because of lack of consensus recommendations and guidelines the management of spilled gallstones vary widely between institutions and individual surgeons. Therefore, the need for further evaluation of the accumulated evidence is needed urgently.

The aim of the present study was to evaluate the evidence of the complications rate of the spilled gallstones overtime by conducting a systematic review.

## Methods

### Literature search strategy

From 2000 until today a literature search was performed in Embase, Medline (Pubmed), Cochrane library, Google scholar, and National Institute for Health and clinical Excellence (NICE) databases using free and MeSH terms (spilled, lost gallstones, complications during laparoscopic cholecystectomy, late complications after laparoscopic cholecystectomy, intraabdominal abscess, retroperitoneal abscess, flank abscess, pigment gallstones, cholesterol gallstones). The search strategy was conducted according to the Preferred Reporting Items for Systematic Reviews and Meta-Analyses (PRISMA) [10].

### Study, selection, and inclusion and exclusion criteria

Publications evaluating the complications of spilled gallstones during laparoscopic cholecystectomy were included. Studies referred to open cholecystectomy and editorials without original data were excluded.

### Data extraction and outcomes

Two reviewers (PG and NDA) independently extracted the following data from the included studies: name of authors, country, year of publication, age, gender, indication for laparoscopic cholecystectomy, reference to spilled gallstones, type of lost stones, number of stones spilled, size of lost stones, location of lost stones, presenting symptoms, time of onset of symptoms after the laparoscopic cholecystectomy, complications caused by lost stones and location found, type of reintervention, 90-day perioperative mortality.

## Results

**Fig. 1:**
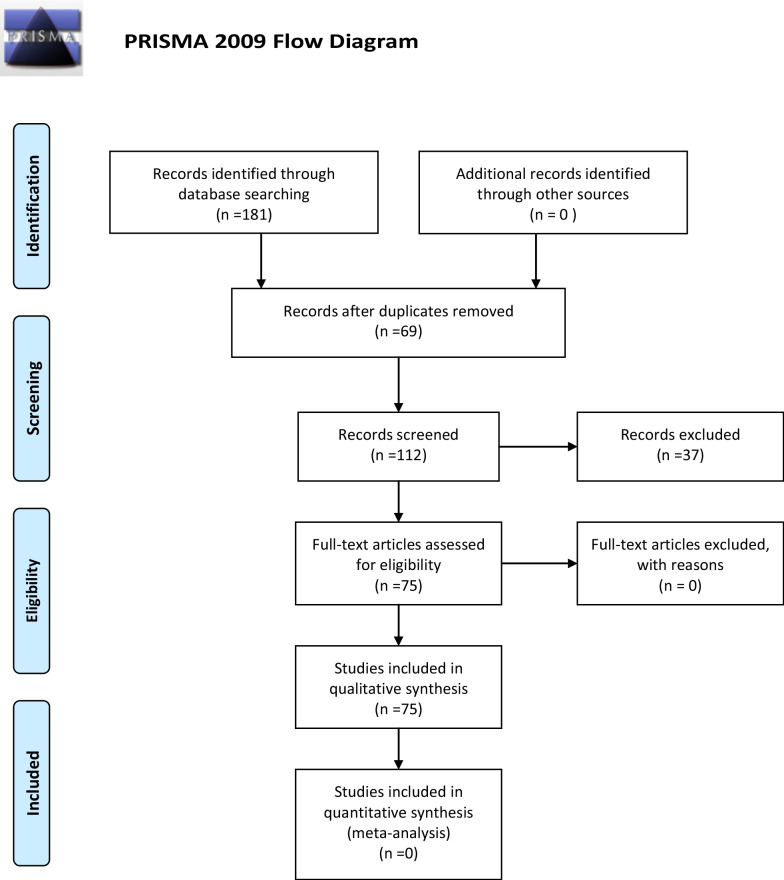
Diagram of the search strategy

Seventy-five articles from a pool of 181 articles were selected including 85 patients [11–85], (Fig. 1, Table 1). The median age of the cohort was 64 years and ranged between 33 and 87 years. The percentage of males and females were of 38% and 62%, respectively. The acute cases were 26(31%). Only 23(27%) of the surgeons reported the perforation of the gallbladder and consequently, spillage of the gallstones in the operative notes. The median time of onset of symptoms was 36 months and ranged between 1 and 180 months; the mode was 24 months. The most common site of lost stones was the right subhepatic, perihepatic, retroperitoneal, right flank and pelvis. Ten (12%) out of 85 cases of lost stones discovered incidentally [18, 19, 23, 39, 47, 49, 52, 58, 71, 75]. Type of lost gallstones discovered during the re-intervention reported by 17 authors(20%), [18–20, 24, 31, 35, 39, 42, 43, 53, 54, 63, 80]; of those 7 (41%) were pigment and 8 (47%) cholesterol gallstones. Seventeen(20%) of authors reported the number of discovered gallstones [18, 25, 27–29, 31, 35, 38, 39, 44, 45, 50, 54, 57, 70, 80, 83]. The size of discovered gallstones was reported by 12(14.11%) authors [18, 19, 25, 28, 31, 35, 37, 52, 63]. The most prevalent presenting symptoms were pain, fever, nausea, vomiting, abdominal swelling, fistula formation, and loss of weight. The most prevalent complications were intrabdominal abscesses 31(36.5%), abdominal wall abscesses 9(10.6%), retroperitoneal abscesses 8(9.4%), thus abscesses in total consisted of 48(56.5%) cases. Notably, 87% of patients underwent a surgical procedure and 12% treated with US ± CT scan guidance drainage, two cases that diagnosed incidentally and were asymptomatic scheduled for regular follow-ups (Table 1). One patient died on the 11th postoperative day after lung decortication for thoracic empyema secondary to lost gallstones [15].

**Table 1:** Study characteristics of the publications for the complications of the spilled gallstones

Author, country, year	Number of patients	Age	Indication for LC	Reference to the spilled stones	Presenting symptoms	Time of onset of symptoms after LC	Complications caused by Lost stones and location found	Type of reintervention
Ray S India, 2021	1	48	NR	NR	Low-grade fever and swelling on the site of the axillary port	39 months	Tender swelling on the site of the axillary port	Surgical removal
Mehmood UK, 2021	1	65	Symptomatic cholelithiasis	Yes	Long standing dry cough, fever and painful swelling over the back in the right paraspinal area	8 years	Large abscess in the right paraspinal region and retroperitoneal abscess	I + D
Guruvaiah USA, 2021	1	61	Acute cholecystitis	Yes	1-year history of intermittent RUQ pain, recurrent bronchitis and pneumonia with mucopurulent cough and sputum since his LC	Recurrent pneumonia since his LC	Bronchobiliary fistula	Trans-diaphragmatic takedown of the BBF and right hepatic middle lobe wedge resection
Djelassi Belgium, 2021	1	82	Perforated necrotic cholecystitis	NR	Chronic fistula at the RUQ	8 years	Abscess between the right internal oblique and transverses abdominis	Fistulectomy and drainage
Tchercansky Argentina, 2020	1	69	Gallbladder empyema	Yes	Loculated pleural effusion of the Right Hemithorax in posterior cost-diaphragmatic recess	5 months	Pleural effusion	CT guided thoracic drainage initially and then Lung decortication by Video Assisted Thoracoscopy
Kafadar Turkey, 2020	1	42	NR	NR	Painful swelling in suprapubic region persistent for 3 days	10 years	Omental granuloma	Partial omentectomy
Marçal Portugal, 2020	1	79	Symptomatic cholelithiasis	NR	Emergent admission for Right subcutaneous lumbar abscess 10 cm with no retroperitoneal extension and renal involvement	3 years	Right subcutaneous lumbar abscess	Surgical drainage
Bolat Turkey, 2020	1	62	Acute Cholecystitis	NR	Incidental finding in the right inguinal hernial sac	5 months	Incidental finding in the right inguinal hernial sac	Surgical excision
Heywood Australia, 2019	1	70	Emergency LC	NR	Incidental finding in the right inguinal hernial sac	5 years	Incidental finding in the right inguinal hernial sac	Surgical excision
Cummings USA, 2019	1	70	Emphysematous cholecystitis and liver abscess	Yes	vague abdominal discomfort	2 years	Sub hepatic	Surgical exploration + drainage
Akhtar Pakistan, 2018	1	78	NR	NR	Recurrent bouts of abdominal pain and fever for the previous 2 weeks in the RUQ	10 years	19 cm Right subdiaphragmatic and retroperitoneal abscess	CT-guided drainage
Tyagi USA, 2018	1	70	Acute Cholecystitis	Yes	Septic shock CT scan: two partly calcified soft tissue masses associated with the right iliopsoas and obturator internal muscles	2 months	Iliopsoas abscess and periprosthetic hip infection	Surgical drainage
Capolupo Italy, 2018	1	73	Chronic cholecystitis	Yes	Peritoneal nodule detected during FU for kidney stones	16 months	Peritoneum, NO complications	Laparoscopic excision
Urade Japan, 2018	1	68	Gangrenous Cholecystitis	Yes	CT findings of omental abscess and ascites around the spleen	7 months	Omental abscess	Laparoscopic partial omentectomy
Ologun 2018	1	52	Biliary colic	NR	Occasional postpranding epigastric pain	4 years	Calcified intraabdominal mass within the omentum detected in routine FU for Lap sleeve gastrectomy	Laparoscopic resection of the mass
Stroobants Belgium, 2018	1	72	Symptomatic cholelithiasis	NR	Intermittent complains for RUQ pain	NR	Subhepatic abscess	Open drainage
Kaplan Israel, 2018	1	73	NR	NR	Six months vague RUQ pain	10 years	Perihepatic abscess	Lap drainage
Kaplan B 2018	1	41	NR	NR	One-month vague RUQ pain	3 years	Perihepatic abscess	Lap drainage
Koichopolos Canada, 2017	1	80	NR	NR	Gastric outlet obstruction, 30 pounds weight loss, progressively worsening nausea, vomiting and significant gastroesophageal reflux	NR	Intramural obstruction of pylorus	Billroth II Distal gastrectomy
Canna UK, 2017	1	79	Chronic cholecystitis	NR	Painful and firm mass on the right flank	5 years	Retroperitoneal abscess	Surgical drainage
Lentz USA, 2017	1	57	Symptomatic cholelithiasis	NR	Cough and right flank pain	2 years	Perihepatic, pulmonary and renal abscesses	Thoracic drainage
Faour Syria, 2017	1	44	Symptomatic cholelithiasis	NR	Mass in the RUQ associated with pain, nausea and early satiety for the last 6 months	6 years	Intra-abdominal cystic mass	Surgical excision
Ragozzino Italy, 2016	1	63	Chronic cholecystitis	NR	Intermittent vague discomfort of RUQ	2 years	Subphrenic abscess	3 × 3 cm mass excised
Kim Korea, 2016	1	59	NR	NR	Constant RUQ pain	5 months	Retroperitoneal mass	5 × 5 cm retroperitoneal mass was excised
Goodman USA, 2016	1	87	Acute Cholecystitis	NR	Right flank pain and tenderness	4 years	Right flank soft tissue tumour	Surgical excision
Moga Romania, 2016	1	66	Acute Cholecystitis	NR	Fever and large abscess in the right lumbar region	4 years	Right lumbar region abscess and subhepatic abscess	Lap drainage
Bedell USA, 2015	1	41	Symptomatic cholelithiasis	NR	Dysmenorrhea progressed to chronic pelvic pain unrelated to menses	9 years	pelvic abscess	Lap drainage
Binagi USA, 2015	1	58	Symptomatic cholelithiasis	NR	Continuous but waxed and waned pain, reaching levels eight out of ten of Likert scale	3 years	Perihepatic abscess	Lap drainage
Grass Switzerland, 2015	1	75	Acute cholecystitis	NR	Periumbilical redness and tenderness	3 years	Abdominal wall abscess in the periumbilical port site	Drainage
Noda Japan 2014	1	52	Symptomatic cholelithiasis	NR	Incidental US finding during medical check up	7 months	Sub hepatic abscess	Percutaneous abscess drainage
Noda Japan, 2014	1	41	Symptomatic cholelithiasis	NR	RUQ pain	13 months	A rounded mass in the subhepatic space	Open drainage
Ahmad UK, 2014	1	37	Symptomatic cholelithiasis, incidental pT1a gallbladder cancer	Yes	Recurrent pain two year after LC	2 years	Multiple tumour embedded gallstones on the diaphragm and lesion in segment VI of the liver	Surgical excision of diaphragmatic nodules and liver segmentectomy VI
Lee Korea, 2013	5	65/55/48/72/80	1.recurrent ac ch/tis 2. Gangrenous Ch/tis 3. Recurrent ac ch/tis 4. Gangrenous ch/tis 5. Recurrent ac ch/tis	Yes	NR	7/18/31/4 months 2nd post day	Subhepatic abscess/cul de sac abscess/umbilical fistula/portal fistula/peritonitis	Drainage/drainage/prolonged wound care/antibiotic administration
Morris USA, 2013	1	71		NR	Pulmonary complains of diffuse abdominal pain, associated with nausea and emesis lasted for 24 h	15 years	Dense mesenteric cicatrix causing ileocolic torsion and cecal volvulus	Ileocecectomy
Peravali UK, 2013	1	61	Acute Cholecystitis	Yes	12-month history of persistent RUQ pain, 8 KG weight loss, anorexia, night sweats, intermittent pyrexical episodes	3 years	Sub hepatic abscess	Lap drainage
Peravali UK, 2013	1	86	Acute cholecystitis	Yes	Chronically discharged right back fistula	5 years	Subphrenic abscess with atmospheric fistula	Lap drainage
Dobradin 2013	1	82	Elective cholecystectomy	NR	RUQ pain lasting for 2 months	8 years	Right flank abscess	I + D
Chatzimavroudis Greece, 2012	1	72	Symptomatic cholelithiasis	Yes	High fever, chills and constant pain in the Right lumbar region for 2 days	6 months	Retroperitoneal abscess	CT-guided drainage
Gorospe Spain, 2013	1	63	Acute Cholecystitis	NR	Fever, malaise, weight loss	6w	Fever of unknown aetiology	NR
Anrique Chile, 2013	1	60	NR	NR	Incidental finding during Lap Gynaecologic procedure	14 years	Multiple gallstones incrusted in the Douglas’ pouch	Surgical removal
Arai ColonJapan, 2013	1	65	Symptomatic cholelithiasis	NR	Referred by GP for further investigation of an abnormal liver mass	4 years	Subphrenic abscess	Wedge resection of the liver and diaphragm
Papadopoulos Greece, 2012	1	86	Symptomatic cholelithiasis	NR	Incidental finding during right hemicolectomy	8 years	Gallstones embedded in the omentum	Removal during right hemicolectomy
Singh USA, 2012	1	42	NR	NR	Worsening Right-sided tenderness and pain, low grade fever, night chills	7 years	50 pounds weight loss over 5 months	Surgical excision of 4 × 6 cm
Rammohan India, 2012	1	50	NR	NR	Minimally painful, slow progressing mass in the RUQ for the last two years	4 years	10 × 5 cm organised extrahepatic mass in the sub-diaphragmatic space extending onto the soft tissues of parietal wall	Laparoscopic piecemeal excision
Kayashima Japan, 2011	1	57	Acute cholecystitis	Yes	Incidental abdominal US showed 3 liver lesions	3 years	Inflammatory pseudotumour of the liver	Posterior segmentectomy and concomitant resection of the diaphragm
Hussain Saudi Arabia, 2010	1	33	Acute cholecystitis	Yes	Intermittent attacks of pain RUQ, nausea, vomiting for 7 months	9 years	Discharging abdominal wall abscess extending to the retroperitoneum	I + D
Pottakkat India, 2010	1		Symptomatic cholelithiasis	NR	Fever, malaise	11 years	Dumbbell abscess in the perihepatic area	Open drainage
Bouasker Tunisia, 2010	1	57	Acute cholecystitis	NR	RIF painful swelling	8 years	Subcutaneous collection	I + D
Gooneratne New Zealand, 2010	1	54	Acute cholecystitis	NR	Recurrent urinary tract infections	14 years	Colovesical fistula	Surgical repair of the fistula
Helme 2009	1	77	NR	NR	Night sweets, right back pain and loin swelling for 2 weeks	5 years	Complex subphrenic, subhepatic and subcutaneous abscesses	US-guided drainage. Patient declined operation to remove the offending gallstones
Morishita Japan, 2009	1	67	Symptomatic cholelithiasis	NR	Incidental finding during FU for aneurysm	1 year	Granuloma	Conservative treatment
Dasari UK, 2009	1	67	Acute cholecystitis	Repeat laparoscopy for septicaemia and drainage of fluid collection	Recurrent lower abdominal pain	2 years	Nodules mimicking peritoneal metastases	Lap excision
Maempel UK, 2009	1	42	Symptomatic cholelithiasis	NR	Strangulated recurrent paraumbilical hernia	10 years	Abdominal wall abscess	I + D
Hougård Denmark, 2008	1	64	Acute cholecystitis	Yes	Referred for Management of abdominal fistulas	7 years	Atmospheric fistula	Surgical excision
Arishi Saudi Arabia, 2008	1	45	Symptomatic cholelithiasis	NR	Central colicky abdominal pains and swelling lasted for 6 months	15 years	Cystic mass of the rectus abdominis	Surgical excision
De Hingh the Netherlands, 2007	1	41	Acute Cholecystitis	Yes	NR	1 year	Rectovaginal pouch abscess	Surgical excision
Stupak USA, 2007	1	72	NR	Yes	Fever, nausea, anorexia, and pain in the RUQ lasting for 3 weeks	11 years	Subhepatic collection	US-guided percutaneous drainage
Pantamowitz USA, 2007	1	53	Symptomatic cholelithiasis	NR	Pelvic pain	7 years	Left overs granuloma	Surgical excision
Wehbe Australia, 2007	1	80	Symptomatic cholelithiasis	NR	Abdominal pain, nausea, diarrhoea	10 years	Mass in the right lower quadrant	Lap excision
Wittich USA, 2007	1	42	Symptomatic cholelithiasis	NR	Severe metrorrhagia, dysmenorrhea	13 months	Abscess in the pouch of Douglas	16 gallstones discovered after transvaginal hysterectomy for severe dysmenorrhoea and metrorrhagia
Bhati UK, 2006 A	1	52	Symptomatic cholelithiasis	NR	Upper abdominal pain	1w	CT: cystic mass in the left lobe of the liver	Open drainage
Bhati UK, 2006 B	1	60	Symptomatic cholelithiasis	NR	Fever and pain in her back	28 months	Subdiaphragmatic abscess	Open drainage
Bhati UK, 2006 C	1	56	Symptomatic cholelithiasis	NR	Fever and pain of the upper abdomen	7 years	Subdiaphragmatic abscess	I + D
Ianniti USA, 2006	1	70	Symptomatic cholelithiasis	NR	Generalised aches and pains	18 months	Subphrenic + pleural abscess	Open and US guided drainage, due to recurrence open removal
Hand USA, 2006	1	50	Biliary pancreatitis	NR	Pain, fever, large fluctuant mass lateral to umbilicus	2 years	Abdominal wall cystic mass	US-guided drainage, later local exploration and excision of the abscess
Viera Italy, 2006	1	72	Symptomatic cholelithiasis	NR	Fever, general malaise and weight loss	18 months	3 inflammatory lesions in seg II and VII	Open excision
Viera Italy, 2006	1	70	Acute Cholecystitis	Yes	Patient asymptomatic, incidental US finding	2 months	Asymptomatic	Watch and see approach
AlSamkari USA, 2004	1	36	Symptomatic cholelithiasis	Yes	Diffuse abdominal pain nausea, vomiting and weakness	11 years	Necrotic transverse colon from mid-ascending to just distal the splenic flexure	Surgical excision
Koç Turkey, 2004	1	75	Symptomatic cholelithiasis	NR	NR	6 years	Retroperitoneal abscess	Percutaneous drainage
Stevens, 2003	1	68	Gallstone pancreatitis	NR	30-pound weight loss and acholic stools	1 year	Subhepatic abscess	Open drainage
Aspelung Iceland 2003	1	NR	NR	NR	Incidental finding during routine hernioplasty	days 10	Gallstones in the hernial sac	Removal during hernia repair
Papasavas Greece, 2002	1	77	Symptomatic cholelithiasis	Yes	Fever, pain	15 months	Right flank abscess	Surgical removal
Yadav, 2002	1	NR	Symptomatic cholelithiasis	NR	NR	1 year	Subphrenic abscess	Open drainage
Van Mierlo, 2002	1	48	Symptomatic cholelithiasis	Yes	Pain in the RUQ, nausea, vomiting	2 years	Subhepatic abscess	Open drainage
Hawasli, 2002 A	1	75	Symptomatic cholelithiasis	NR	Pain, fever	4 years	Abdominal wall abscess	Open drainage
Hawasli, 2002 B	1	43	Symptomatic cholelithiasis	NR	Pain, fever	2 months	Subdiaphragmatic and subhepatic abscesses	
Famulari, 2002	1	NR	Symptomatic cholelithiasis	NR	Dysuria, pollakiuria, vesical tenesmus	2 years	Urinary bladder granuloma	Partial cystectomy
Werber USA, 2001	1	64	Symptomatic cholelithiasis	Yes	Low-grade fever with chills, night sweats, weight loss, fatigue	1 month	Sub hepatic abscess and 3 cm round mass with speculated borders in the right lower lobe of the lung	Right thoracotomy
Yao China, 2001	1	NR	Symptomatic cholelithiasis	NR	NR	2 years	Periumbilical abscess	Surgical excision
Battaglia Italy, 2001	1	39	Symptomatic cholelithiasis	NR	Fever and pain	9 years	Abdominal wall abscess	Surgical excision
Ok E Turkey, 2000	1	NR	Symptomatic cholelithiasis	NR	Umbilical port site hernia	3 months	Incisional umbilical port site hernia	Surgical excision
Bebawi, USA. 2000	1	56	Chronic cholecystitis	Yes	Incidental finding	2 months	Gallstones in the hernial sac	Removed during hernia repair
Total	85 cases	64(33–87)	Acute cases 26(31%)	23 authors (27%)	Most prevalent Fever and pain	36 months (1–180)	TA: 48(56.5%) IAA:31(36.5%) RPA: 8(9.4%) AWA: 9(10.6%) IF:10(11.8%)	Open procedure 61 (72%) Lap procedure: 13 (15%) US or CT drainage: 9(11%) 2 watch and see approach

*IAA* intraabdominal abscesses, *RPA* retroperitoneal abscesses, *AWA* abdominal wall abscesses, *TA* total abscesses, *IF* incidental findings: incidental findings, *Seg* segment, *LC* laparoscopic cholecystectomy, *NR* nonreported

## Discussion

Complications of the spilled gallstones can be described under the umbrella eponym, disease of the medical progress DOMP. There is a contrasting difference with the open cholecystectomy; because spillage of gallstones during open cholecystectomy is more easier identified and retrieved there are very few reports with the above complication [86, 87].

At the present 96% of all cholecystectomies are performed laparoscopically [88]. In general, the characteristics of the cohorts of patients who underwent laparoscopic and open cholecystectomies differ essentially. The laparoscopic cohort is consisted of younger and healthier patients whereas the open cohort tend to be older, less well, and generally the open cholecystectomy is performed in higher-risk patients [89, 90]. Another important characteristic of the laparoscopic era is the broadening of the indications and the dramatic increase in the number of LCs performed for acalculous disease [91].

Taking into account that present studies reported that older age is a predicting factor for developing complications following spillage of gallstones [3]. We can see a controversy with the above evidence that demonstrates that the LC cohort includes younger and healthier patients. Therefore, there is a strong indication for further investigation and identification of the co-factors (e. g comorbidities, type of gallstones, acute vs chronic cases) that predispose to above complication. In the present study the median age was 64 years and varied widely from 33 to 87 years (Table 1).

An analysis performed at American College of Surgeons-National Surgical Quality Improvement Program hospitals found the rates of severe morbidity of laparoscopic and open cholecystectomy to be 1.4% and 11.1%, respectively [92].

Notably, the Swiss Association of Laparoscopic and Thoracoscopic Surgeons (SALTS) database defined the rates following LC for patients only with diagnosis of acute or chronic cholecystitis for intraoperative complications at 7%, postoperative local complications at 4%, and postoperative systemic complications at 2.3% [93]. The above comparison demonstrates that when the investigation is further focused to acute vs chronic cases the incidence rate of severe morbidity of LC increases dramatically from 1.4 to 7% [92, 93]. Although, it is reported that the acute cholecystitis is predisposing factor for complications of spilled gallstones [3]; in the present study only 31% were the acute cases. Therefore, future studies need to shed further light on the above topic. Moreover, in the present study, only 23(27%) of the surgeons reported the incidence of gallbladder perforation and spillage of gallstones in the operative notes. Furthermore, Mullerat et al. reported that only one fifth of the surgeons included in the consent form spillage of the gallstones as a potential complication. In addition, if this occurred during the operation they do not report it to GPs and patient because they consider it an innocent complication; although this information is going to help colleagues to resolve future diagnostic dilemmas [9].

Although, it is reported that the number of spilled gallstones more than 15, size > 1.5 cm and pigment type gallstones are predicting factors of complications of spilled gallstones [3]; in the present study, type of lost gallstones, number, and size of discovered gallstones during the re-intervention reported at 17(20%), 17(20%), and 12(14,11%), respectively. In particular, pigmented and cholesterol gallstones consisted 41% and 47%, respectively. Therefore, future studies should be more meticulous regarding describing type, size, and number of discovered gallstones because the accumulated information will further help describing in details the predicting risk factors and furthermore, this will help in the standardisation of the management of spilled gallstones.

The median time to onset of symptoms was 36 months and ranged from 1 to 180 months. It is obvious that was ranged widely. Considering the above finding and the widely ranged follow-up, the results of the incidence rates of complications should be treated cautiously because time, follow-up, and institutions bias might have influenced the results.

Notably, vast majority of the cases of undiscovered gallstones required open intervention. In particular, 61(72%) patients underwent open surgery and 13(15%) patient laparoscopic procedure, 9(11%) treated either with US and/or CT scan guided drainage. Two cases that detected early postoperatively and were asymptomatic scheduled for regular follow-ups. These finding underlines that although the incidence rate of the complications is low when they become symptomatic the treatment of choice is surgical intervention Therefore, there is urgent need for standardisation and clinical guidelines for the management of spilled gallstones.

### Limitations

The results of the present study should be treated cautiously because all the included studies were case reports. Therefore, institutional, national, underpowered sample size, learning curve, performance and follow-up bias might have influenced the results. Another topic that needs special attention is the incidence rate. Usually, the cases with most complicated presentation and with worst outcomes published as case reports. On the contrary cases with mild symptoms and better outcomes, usually are not publishable. Therefore, an international registry and audit may help to define precisely the incidence rate, and severity of complications of spilled gallstones.

## Conclusions

The current evidence demonstrates that although the incidence rate of complications varies widely the majority of the patients demonstrated severe morbidity and required surgical interventions. Therefore, urgent standardisation of the management of spilled gallstones is needed. Surgeons must document all cases of spilled stones in the operative notes. Moreover, GPs and patients should be informed about the incidence, this will help to resolve diagnostic dilemmas in the future. Hospitals should review their policy by conducting audits and surgical societies should use the above information and national databases in order urgently to formulate clinical guidelines.

## Data Availability

The authors declare that data supporting the findings of this study are available within the article.
